# Comparison of Health Complaints, Occupational Risks, and Occupational Health Practices of Healthcare Workers According to Professions and Departments in the Hospital

**DOI:** 10.7759/cureus.65614

**Published:** 2024-07-29

**Authors:** Ayse Betul Taskingul, Sibel Kiran, Esra Emerce

**Affiliations:** 1 Department of Pharmaceutical Toxicology, Faculty of Pharmacy, Gazi University, Ankara, TUR; 2 Department of Public Health, School of Medicine, Koc University, Istanbul, TUR

**Keywords:** occupational exposure, hospital, workplace safety, chemical toxicants, occupational health and safety, healthcare workers

## Abstract

Background: It is essential to protect the health of healthcare workers who constitute a large part of the workforce worldwide and whose importance has become more evident after the recent pandemic. There are numerous occupational hazards for healthcare workers in hospitals.

Purpose: The study aims to assess the exposure hazards of healthcare workers and their health complaints, as well as their awareness, knowledge, opinions, and attitudes towards occupational health and safety (OHS), considering workers’ professions and departments in a public hospital. This cross-sectional study conducted a survey among healthcare workers (n=608) who worked at Yozgat City Hospital, Yozgat, Turkey.

Results: The majority of workers were nurses (43.4%, N=264/608). Latex exposure (63.7%, N=387/608) and noise (55.8%, N=339/608) were the most common exposed hazards, and the risk varies depending on their professions and job descriptions. However, the risk perception of workers was priorities of infectious diseases (48.5%, N=292/602) and violence (27.4%, N=165/602). Musculoskeletal system problems (71.9%, n=439/608) were observed very frequently in workers. Additionally, 9.2% (N=56/608) of workers were diagnosed with an occupational disease. The unit and profession most commonly diagnosed with occupational diseases were the laboratory (22.5%, N=9/40) and midwives (19.4%, N=14/72), respectively. The frequency of workers who stated that they had a work accident at least once in their lives was 31.9% (N=194/608), and higher frequencies belonged to nurses, health officers, and midwives. Additionally, the emergency department was the riskier unit. The study conducted relationship analyses between exposure to various occupational agents at different exposure frequencies and various health complaints. The relationships of occupational hazards such as chemotherapeutics, anesthetic gases, aerosol type drugs, sterilization and disinfection agents, xylene, toluene, formaldehyde, and surgical smoke with health complaints such as liver, dermal diseases, respiratory problems, and varicose veins have been determined.

Conclusions: The hospital workers had a high rate of injuries to sharp objects and musculoskeletal systems. Remarkably, operating rooms and emergency rooms were found to be riskier in terms of work accidents. More than half of healthcare workers may delay using personal protective equipment (PPE) due to excessive workload. Further studies are needed on the effects of more specific occupational chemicals and diseases, such as varicose veins, fertility, and neurological problems. Frequent risk assessments, effective training, workload reduction, and biomarker monitoring are crucial for hospital workplace safety.

## Introduction

Within the scope of occupational health and safety (OHS), activities and strategies have been designed and continue to be developed today to predict, identify, evaluate, and control hazards that may harm the health and well-being of workers in the workplace. Despite all these efforts, it can be seen from the data of recent years that accidents, diseases, and negative health problems among workers still continue. According to the latest report of the International Labor Organization (ILO), 1.88 million people worldwide died from occupational risk factors in 2016; 80.7% of work-related deaths were diseases, and 19.3% were injuries [1]. In the work-life-health relationship, sector-specific evaluations are important. In the health sector, where health protection, treatment, rehabilitation, and support services are provided, healthcare workers constitute 65.1 million of the global labor force in 2020 [2]. Although healthcare workers are considered a single group, since healthcare services are provided by people from different departments and various professions, the workers' professions, job descriptions, and the units they work, as well as workplace environment factors, may also differ. WHO classifies healthcare professionals largely on the International Standard Classification of Occupations (ISCO-08). Although numbers vary in many countries based on the WHO National Health Workforce Accounts (NHWA) data portal, there are 1,350,528 healthcare workers in Turkey. Of these, 194,688 were physicians, 42,359 were dentists; 38,981 were pharmacists; 243,565 were nurses; 59,632 were midwives; 232,661 were other health personnel; and 538,642 were other personnel and service personnel [3].

Hospitals are workplaces that host healthcare workers working in various fields, employ different techniques and practices, contain many hazards, and are classified as "very dangerous" workplaces in terms of OHS. In EU countries, the manufacturing sector ranks first in non-fatal accidents at work with 19.2%, while the health and social services sector ranks second with 13.5% [4]. In 2011, 253,700 work-related injuries and illnesses were recorded in United States (US) hospitals. Nearly three times as many injuries have been reported to occur in hospitals compared to professional and business services, which include a variety of traditional white-collar jobs [5]. While performing their jobs, healthcare workers may be exposed to physical factors such as noise and radiation, ergonomic factors, violence, and stress, especially infections that cause contagious diseases. In hospitals, other dangers that need to be taken seriously are the presence of chemicals, including antineoplastic drugs, aerosolized drugs, strong disinfectants, anesthetic gases, surgical smoke, chemical sterilization, and laboratory chemicals. In 1988, the National Institute for Occupational Safety and Health (NIOSH) stated the dangers and risk factors faced by healthcare workers in hospitals as 29 types of physical, 25 types of chemicals, 24 types of biological, 10 types of psychosocial, and six types of ergonomics factors. However, as time passes, the dangers in hospitals are changing with the development of the chemical industry, the emergence of new infectious diseases, the updating of hospital equipment and environmental designs due to the advancement of technology, and social and cultural changes.

In terms of occupational health, determining the prevalence of exposure to various agents at the level of the profession and the units they work in and examining the relationship between possible health problems can contribute to revealing occupational risks for those working in hospitals where many chemicals and physical and biological agents are present. This study aimed to determine the nature and frequency of exposure to hazards that healthcare workers working in public hospitals may be exposed to evaluate their current diseases and complaints, as well as their awareness, behaviors, and knowledge of OHS practices.

## Materials and methods

This cross-sectional study was conducted at the Turkish Ministry of Health Yozgat City Hospital, a public hospital. The hospital is the largest hospital in the province, with a closed area of 142,000 m^2^ and a registered bed capacity of 475, out of two hospitals located in the Central district of Yozgat province, which has a population of 418,442 in the Central Anatolia Region. Ethical permission was received from the Gazi University Ethics Commission (23.02.2023, No:03). In addition, the approval of the Yozgat Provincial Health Directorate Scientific Research Commission was obtained (23.02.2023 No: E-16180230-772.02-209941378). A questionnaire consisting of 41 structured and open-ended questions was prepared by the researchers based on the research hypothesis and literature. The questionnaire prepared as a data collection tool was added to the digital environment using the "Google Forms" application. A preliminary study was conducted on five healthcare professionals to evaluate whether the questionnaire could be applied in a healthy way. The data were collected over a two-month period between March 15 and May 15, 2023. The research was planned to reach all employees without selecting a sample, and the participation of at least 70% of each group of healthcare workers was aimed to increase the power of the data. A total of 608 healthcare professionals participated in the research, and 72.3% (N=841) of the entire population was reached. A questionnaire was administered to healthcare professionals working in the hospital who read the informed consent form and agreed to participate in the study.

In the study, healthcare professionals were grouped according to their professional titles and the unit in which they worked. The reason for this is that there are healthcare personnel with different titles working in the same unit, as well as healthcare personnel with the same title but different job descriptions due to being in different branches. Therefore, it was assumed that they would be exposed to similar workplace environments within similar workplace environments and tasks. According to professional titles, participants were grouped as doctors, midwives, nurses, health officers, health technicians, and others (pharmacist, dietitian, perfusionist, psychologist, physiotherapist, audiologist, occupational therapist, and child development specialist). On the other hand, regarding the working unit, participants were grouped as working in the emergency room, operating room, laboratory, radiology, service, intensive care unit, and others (pharmacy, medical consumables warehouse, audiology unit, dietitian unit, respiratory function test measurement unit, EEG measurement unit, quality management unit, patient rights unit, breastfeeding unit, child and adolescent mental health unit, etc.)

Statistical analysis

Data were analyzed with Statistical Product and Service Solutions (SPSS; version 22.0; IBM SPSS Statistics for Windows, Armonk, NY). Descriptive statistics for categorical data were given as frequency (%), and for numerical data, it was given as mean, standard deviation, minimum, and maximum value. Categorical data were presented using cross-tables, and difference analyses in terms of frequencies between groups were evaluated using the chi-square test or Fisher's exact test, depending on whether they met the assumptions. Possible factors identified in multivariate analyses were examined using logistic regression analysis. The Hosmer-Lemeshow test was used to determine the model fit. Evaluations of the association between complaints and exposure to occupational hazards were made based on the odds ratio (OR) and the relevant 95% confidence interval values. In all analyses, it was considered statistically significant when the calculated p-value was less than 0.05.

## Results

Descriptive characteristics of the participants and evaluations of their health conditions

When the descriptive characteristics of the participants were evaluated (Table 1), 64.1% (N=390/608) of the healthcare workers were women, and the majority of the participants were between the ages of 26-35 (40%, N=244/608) (p<0.001). The distribution of healthcare professionals varies according to their profession (p<0.001), and the majority of those working in the hospital are nurses (43.4%, N=264/608). Additionally, 29.4% (N=179/608) of the participants were in the wards, 17.3% (N=105/608) in the operating rooms, 14.8% (N=90/608) in the emergency department, 12.5% (N=76/608) in intensive care units, 16.9% (N=103/608) in other departments, 6.6% (N=40/608) in laboratories, and 2.5% (N=15/608) in radiology units.

**Table 1:** Demographic characteristics of the health workers (n=608) ¥: Pharmacist, physiotherapist, dietitian, psychologist, child development specialist, audiologist, perfusionist, occupational therapist The data have been represented as n and %. p<0.05 is considered significant. * NPar test; ** Pearson chi-square test

Characteristics		n (%)	p
Sex	Female	390 (64.1)	<0.001*
	Man Total	218 (35.9) 608 (100)	
Age	18-25	43 (7.1)	<0.001** (Χ^2^: 139.80)
	26-35	244 (40.1)	
	36-45	180 (29.6)	
	46 ≤ Total	141 (23.2) 608 (100)	
Education	High school	33 (5.4)	<0.001** (Χ^2^: 407.87)
	University	428 (70.4)	
	M.Sc./Ph.D./Proficiency Total	147 (24.2) 608 (100)	
Profession	Doctors	104 (17.1)	<0.001** (Χ^2^: 341.72)
	Nurses	264 (43.4)	
	Midwives	72 (11.8)	
	Health officers	83 (13.7)	
	Health technicians	47 (7.7)	
	Others^¥^ Total	38 (6.3) 608 (100)	
Years of working in the profession	≤ 5	152 (25.0)	<0.001** (Χ^2^: 25.32)
	6-15	200 (32.9)	
	16-25	142 (23.4)	
	26 ≤ Total	114 (18.8) 608 (100)	
Work shift	Rotating shift	281 (46.2)	<0.001** (Χ^2^: 293.97)
	Day shift	213 (35.0)	
	Only on-call	113 (18.6)	
	Night shift Total	1 (0.2) 608 (100)	

When the smoking status of healthcare professionals was evaluated, 55.8% (N=339/608) of all participants stated that they had never smoked, 32.1% (N=195/608) were smokers, and 12.2% (N=74/608) were former smokers. Among the professions, the highest smoking prevalence (51.8%, N=43/83) was seen in health officers, with 20.44 ± 7.87 cigarettes/day. It was also observed that those working in the emergency department (46.7%, N=42/90) had the highest smoker frequency. On the other hand, the highest prevalence among non-smokers was in doctors (63.5%, N=66/104). In addition, three healthcare workers smoke e-cigarettes.

Additionally, 22.9% (N=139/608) of the participants reported having a diagnosed chronic disease. It was observed that the highest frequency of chronic diseases was among midwives (36.1%, N=26/72), and the second was among health officers (30.1%, N=25/83) (p<0.001, Χ2: 20.53). The ‘other’ group of professions had the lowest frequency of chronic diseases (5.3%, n=2/38). No significant relationship was found between having a chronic disease according to the working unit studied (p=0.150, Χ2: 9.44). It was determined that there was a statistically significant difference in the frequency of chronic diseases according to the years of work in the profession and age (p<0.001, Χ2: 61.69). To determine the factors that increase the risk of chronic diseases, the effects of age, sex, profession, years of working in the profession, unit worked, and smoking status were examined by multivariate analysis. Among the independent predictors examined in predicting the risk of chronic disease, only the age groups 36-45 (p=0.037) and ≤ 46 (p=0.016) were found to be significant. The most common chronic disease among all healthcare workers is hypertension (23.6%, N=35/206), and thyroid-related diseases (20.9%, N=31/206) and lung diseases (20.3%, N=30/206) are also frequently observed.

Specifically, 9.2% (N=56/608) of all healthcare workers reported that they were diagnosed with an occupational disease. It was determined that the most common occupational diseases were in midwives (19.4%, N=14/72) and health officers (15.7%, N=13/83). No occupational disease was reported in the professions grouped as ‘other’. The units most frequently diagnosed with occupational diseases were the laboratory (22.5%, N=9/40), operating room (12.4%, N=13/105), and emergency department (12.2%, N=11/90) (p=0.008, Χ2: 17.27). The diagnosis of occupational disease was most common in people who had been working for more than 26 years (19.3%, n=22) and in those over 46 years of age (18,4%, N=26/141). There was no significant difference between gender and occupational diseases (p=0.233, Χ2: 1.42). However, when the effect of age, profession, years of working in the profession, and the unit worked were examined with multivariate analysis, none of the predictors examined in predicting the risk of occupational disease made a significant difference (p>0.05).

The prevalence of blood-borne diseases among all healthcare workers was determined to be 1.6% (N=10/608). Moreover, 3.1% (N=19/608) of the workers did not want to indicate whether they had a blood-borne disease. While 60% (N=6/10) of the patients did not know the source of the contamination, only 10% (N=1/10) stated that the contamination occurred during professional practices. 30% (N=3/10) reported that they contracted the infection through non-professional means.

It was found that 31.9% (N=194/608) of the participants had at least one work accident or injury during their working lives (Table 2). While the frequency of occupational accidents/injuries among health workers is higher among nurses, health officers, and midwives (p<0,001, Χ2: 21.55), it is also more prominent among those working in the emergency department (p=0.028, Χ2: 14.15). Among all reported causes of work accidents and injuries, soft tissue traumas caused by various reasons (needle sticks/cuts/bruises, etc.) were in first place with 95.4% (N=185/194), and waist, muscle, and joint problems were in second place with 88.7% (N=172/194). Traumas caused by slips, falls, etc. (51.5%, n=100/194), physical violence (33.0%, n=64/194), poisoning (15.5%, n=30/194), and electric shock and burns (14.9%, n=29/194) were the other common causes of accidents and injuries. When the causes of occupational accidents and injuries were evaluated, soft tissue traumas were most frequently experienced by health technicians (41.7%, N=10/24) among the professions. Health officers were the profession that suffered the most from traumas/injuries from slips, falls, etc., with 20.2% (N=19/94). The professional group that most frequently experienced physical violence was doctors, with a frequency of 16% (N=13/81). Among the workers suffering from accidents, electric shock, and burns were most frequently observed in operating rooms (46.0%, n=17/37), physical violence by patients/patients' relatives/staff was most frequently observed in the emergency department (51.3%, N=20/39). Contrary to the frequency (31.9%, N=194/608) of work accidents throughout their entire working lives, 6.7% (N=41/608) of the participants reported having had at least one work accident or injury in the last year at the hospital where the research was conducted.

**Table 2:** Health workers’ occupational accidents and injuries status throughout their working lives and their relationship with some risk factors ¥: Pharmacist, physiotherapist, dietitian, psychologist, child development specialist, audiologist, perfusionist, occupational therapist; #: Pearson chi-square test The data have been represented as n and %. p<0.05 is considered significant.

Variables		Yes n (%)	No n (%)	Total n (%)	p#
Health workers (n=608)		194 (31.9)	414 (68.1)	608 (100.0)	
Profession	Doctors	26 (25.0)	78 (75.0)	104 (100.0)	0.001 (Χ^2^: 21.55)
	Nurses	25 (34.7)	47 (65.3)	72 (100.0)	
	Midwives	100 (37.9)	164 (62.1)	264 (100.0)	
	Health officers	30 (36.1)	53 (63.9)	83 (100.0)	
	Health technicians	11 (23.4)	36 (76.6)	47 (100.0)	
	Others^¥^	2 (5.3)	36 (94.7)	38 (100.0)	
Working unit	Emergency department	39 (43.3)	51 (56.7)	90 (100.0)	0.028 (Χ^2^: 14.15)
	Operating room	37 (35.2)	68 (64.8)	105 (100.0)	
	Laboratory	16 (40.0)	24 (60.0)	40 (100.0)	
	Radiology	3 (20.0)	12 (80.0)	15 (100.0)	
	Services/wards	57 (31.8)	122 (68.2)	179 (100.0)	
	Intensive care unit	19 (25.0)	57 (75.0)	76 (100.0)	
	Others	23 (22.3)	80 (77.7)	103 (100.0)	
Sex	Female	138 (35.4)	252 (64.6)	390 (100.0)	0.014 (Χ^2^: 6.05)
	Men	56 (25.7)	162 (74.3)	218 (100.0)	
Years of working in the profession	≤ 5	35 (23.0)	117 (77.0)	152 (100.0)	0.01 (Χ^2^: 11.33)
	6-15	60 (30.0)	140 (70.0)	200 (100.0)	
	16-25	56 (39.4)	86 (60.6)	142 (100.0)	
	26 ≤	43 (37.7)	71 (62.3)	114 (100.0)	
Work shift	Day shift	57 (26.8)	156 (73.2)	213 (100.0)	0.09 (Χ^2^: 6.48)
	Night shift	1 (100.0)	0	1 (100.0)	
	Rotating shift	94 (33.5)	187 (66.5)	281 (100.0)	
	Only on-call	42 (37.2)	71 (62.8)	113 (100.0)	

When the workers were asked if they had any problems regarding their health, the most common complaint of all healthcare professionals (71.9%, N=437/608) was musculoskeletal system problems. Additionally, 33.1% (N=201/608) of the workers reported that they experienced varicose veins, 27.3% (N=166/608) experienced allergic problems, and 27.1% (N=165/608) reported that they experienced skin-related diseases. Although less frequently, 16.9% (N=103/608) of all healthcare workers have memory problems/neurological problems, 14.5% (N=88/608) have respiratory problems, 6.7% (N=41/608) liver, 5.9% (N=36/608) kidney problems, 5.6% (N=34/608) other, and 2.8% (N=17/608) fertility problems. Among the health problems they complained about, it was determined that there was a significant difference in skin-related diseases (p=0.006), varicose veins (p<0.001), and musculoskeletal system diseases (p<0.001), according to the profession. The occupational groups where skin diseases are most common are nurses (34.5%, N=91/264) and health technicians (31.9%, N=15/47). Forty-two out of 72 (58.3%) midwives and 110 out of 264 (41.7%) nurses complain about varicose veins. The majority of workers complained of musculoskeletal system diseases, especially since 93.1% (N=67/72) of midwives suffered from this condition. Meanwhile, 72.3% (n=191/264) of nurses and 72.3% (N=60/83) of health officers stated that they experienced this problem. Profession groups included in the ‘other’ group (50.0%, N=19/38) were determined to be the group that experienced musculoskeletal system diseases least frequently. When examined in terms of the units worked, the units most frequently experiencing varicose vein problems were those in the other group (42.7%, N=44/103) and those working in the intensive care unit (40.8%, N=31/76) (p=0.005). Complaints that differ significantly depending on gender are fertility, skin, allergy problems, memory/neurological problems, varicose veins, and musculoskeletal system problems (p<0.05), and all these complaints are observed more frequently in women.

Meanwhile, 65.3% (N=397/608) of workers have children; 12.1% (N=48/397) of workers with children stated that their babies were born prematurely, and 0.8% (N=3/397) stated that their babies had congenital defects. Seventy-six out of 255 (29.8%) women with children stated that they had a history of miscarriage at least once in the past. A history of miscarriage (median: 1.5, min-max: 1-6) was reported, with a frequency of 24.6% (N=96/390) among all working women.

Evaluations of healthcare workers' exposure to occupational hazards

Healthcare workers think that they are most frequently exposed to the following hazards in their workplace environments: 48.5% (N=292/602) of infectious diseases, 27.4% (N=165/602) of violence, 22.4% (N=135/602) of injuries with sharp objects, and 15.4% (N=93/602) of exposure to drugs and chemicals. Other occupational dangers, radiation, physical hazards, ergonomic risks, and allergies were also included in the mentioned risks. On the other hand, 3.8% (N=23/602) of workers thought that they had no exposure to any occupational risks. The risks that workers think they encounter most at work vary depending on their profession and the unit they work in (data not shown).

However, when questioned about exposures to the major hazards listed by researchers, real exposure situations were found to be different from what they thought the exposure scenario to dangers. Considering all healthcare workers, latex use (63.7%, N=387/608) and noise (55.8%, N=339/608) were reported as the most frequently exposed hazards (Table 3). It has been observed that there are some differences between professions in terms of exposure to hazards in the hospital environment and that health technicians are generally the riskiest profession. It was reported that 40.4% (N=19/47) of health technicians were frequently exposed to anesthetic gases and ionizing radiation, 38.3% (N=18/47) were frequently exposed to surgical smoke, and approximately 17% of midwives (N=12/72) and health technicians (N=8/47) were frequently exposed to formaldehyde. Wards/services units and intensive care units were the riskier units in the hospital in terms of chemotherapeutics exposure. The workers in the operating room area were the ones who had the most contact with anesthetic gases and surgical smoke, with 50.5% (N=53/105) and 57.1% (N=60/105). The units most exposed to aerosol-type drugs were intensive care units and emergency departments. As expected, the field of work most frequently exposed to ionizing radiation is radiology (60.0%, N=9/15) (p<0.001, Χ2: 218.41). Moreover, 35.6% of those working in emergency rooms (N=32/90) and intensive care units (N=27/76) reported that they were sometimes exposed to radiation.

**Table 3:** The relationship between healthcare worker professions and their exposure to some occupational hazards The data have been represented as n and %. p<0.05 is considered significant.

Chemical, physical, and biological dangers	Exposure	All health workers (N=608, 100%)	Doctor (N=104, 100%)	Midwives (N=72, 100%)	Nurses (N=264, 100%)	Health officers (N=83, 100%)	Health technicians (N=47, 100%)	Other (N=38, 100%)	p#
		n (%)	n (%)	n (%)	n (%)	n (%)	n (%)	n (%)	
Chemotherapeutics	Never	479 (78.8)	87 (83.7)	60 (83.3)	192 (72.5)	71 (85.5)	39 (83.0)	30 (78.9)	0.027 (Χ^2^: 27.22)
	Occasionally	96 (15.8)	14 (13.5)	8 (11.1)	59 (22.3)	7 (8.4)	4 (8.5)	4 (10.5)	
	Often	14 (2.3)	1 (1.0)	3 (4.2)	5 (1.9)	1 (1.2)	1 (2.1)	3 (7.9)	
	No opinion	19 (3.1)	2 (1.9)	1 (1.4)	8 (3.0)	4 (4.8)	3 (6.4)	1 (2.6)	
Anesthetic gases	Never	435 (71.5)	67 (64.4)	61 (84.7)	194 (73.5)	55 (66.3)	23 (48.9)	35 (92.1)	<0.001 (Χ^2^: 79.72)
	Occasionally	92 (15.1)	24 (23.1)	6 (8.3)	46 (17.4)	12 (14.5)	4 (8.5)	0	
	Often	61 (10.0)	11 (10.6)	4 (5.6)	15 (5.7)	11 (13.3)	19 (40.4)	1 (2.6)	
	No opinion	20 (3.3)	2 (1.9)	1 (1.4)	9 (3.4)	5 (6.0)	1 (2.1)	2 (5.3)	
Aerosolized drugs	Never	357 (58.7)	61 (58.7)	53 (73.6)	134 (50.8)	51 (61.4)	28 (59.6)	30 (78.9)	0.068 (Χ^2^: 23.84)
	Occasionally	173 (28.5)	30 (28.8)	12 (16.7)	90 (34.1)	23 (27.7)	13 (27.7)	5 (13.2)	
	Often	55 (9.0)	10 (9.6)	5 (6.9)	30 (11.4)	4 (4.8)	4 (8.5)	2 (5.3)	
	No opinion	23 (3.8)	3 (2.9)	2 (2.8)	10 (3.8)	5 (6.0)	2 (4.3)	1 (2.6)	
Sterilization agents	Never	208 (34.2)	31 (29.8)	22 (30.6)	94 (35.6)	23 (27.7)	17 (36.2)	21 (55.3)	0.083 (Χ^2^: 23.06)
	Occasionally	222 (36.5)	48 (46.2)	22 (30.6)	87 (33.0)	37 (44.6)	16 (34.0)	12 (31.6)	
	Often	163 (26.8)	23 (22.1)	22 (37.5)	75 (28.4)	20 (24.1)	13 (27.7)	4 (10.5)	
	No opinion	15 (2.5)	2 (1.9)	1 (1.4)	8 (3.0)	3 (3.6)	1 (2.1)	1 (2.6)	
Disinfection agents	Never	237 (39.0)	42 (40.4)	26 (36.1)	97 (36.7)	33 (39.8)	17 (36.2)	22 (57.9)	0.019 (Χ^2^: 28.42)
	Occasionally	208 (34.2)	37 (35.6)	27 (37.5)	97 (36.7)	26 (31.3)	11 (23.4)	10 (26.3)	
	Often	130 (21.4)	23 (22.1)	17 (23.6)	59 (22.3)	13 (15.7)	14 (29.8)	4 (10.5)	
	No opinion	33 (5.4)	2 (1.9)	2 (2.8)	11 (4.2)	11 (13.3)	5 (10.6)	2 (5.3)	
Xylene	Never	458 (75.3)	85 (81.7)	53 (73.6)	195 (73.9)	60 (72.3)	32 (68.1)	458 (75.3)	0.619 (Χ^2^: 12.78)
	Occasionally	42 (6.9)	3 (2.9)	5 (6.9)	24 (9.1)	6 (7.2)	3 (6.4)	42 (6.9)	
	Often	16 (2.6)	4 (3.8)	2 (2.8)	6 (2.3)	2 (2.4)	1 (2.1)	16 (2.9)	
	No opinion	92 (15.1)	12 (11.5)	12 (16.7)	39 (14.8)	15 (18.1)	11 (23.4)	92 (15.1)	
Toluene	Never	463 (76.2)	86 (82.7)	53 (73.6)	198 (75.0)	61 (73.5)	32 (68.1)	33 (86.8)	0.779 (Χ^2^: 10.62)
	Occasionally	40 (6.6)	4 (3.8)	5 (6.9)	22 (8.3)	5 (6.0)	3 (6.4)	1 (2.6)	
	Often	13 (2.1)	1 (1.0)	2 (2.8)	6 (2.3)	2 (2.4)	1 (2.1)	1 (2.6)	
	No opinion	92 (15.1)	13 (12.5)	12 (16.7)	38 (14.4)	15 (18.1)	11 (23.4)	3 (7.9)	
Formaldehyde	Never	371 (61.0)	67 (64.4)	39 (54.2)	163 (61.7)	45 (54.2)	24 (51.1)	33 (86.8)	0.02 (Χ^2^: 28.33)
	Occasionally	127 (20.9)	24 (23.1)	19 (26.4)	53 (20.1)	21 (25.3)	9 (19.1)	1 (2.6)	
	Often	63 (10.4)	7 (6.7)	12 (16.7)	27 (10.2)	8 (9.6)	8 (17.0)	1 (2.6)	
	No opinion	47 (7.7)	6 (5.8)	2 (2.8)	21 (8.0)	9 (10.8)	6 (12.8)	3 (7.9)	
Surgical smoke	Never	418 (68.8)	65 (62.5)	50 (69.4)	193 (73.1)	57 (68.7)	21 (44.7)	32 (84.2)	<0.001 (Χ^2^: 53.49)
	Occasionally	89 (14.6)	18 (17.3)	14 (19.4)	37 (14.0)	12 (14.5)	6 (12.8)	2 (5.3)	
	Often	74 (12.2)	19 (18.3)	6 (8.3)	23 (8.7)	7 (8.4)	18 (38.3)	1 (2.6)	
	No opinion	27 (4.4)	2 (1.9)	2 (2.8)	11 (4.2)	7 (8.4)	2 (4.3)	3 (7.9)	
Ionized radiation	Never	362 (59.5)	55 (52.9)	55 (76.4)	153 (58.0)	49 (59.0)	16 (34.0)	34 (89.5)	<0.001 (Χ^2^: 73.92)
	Occasionally	142 (23.4)	26 (25.0)	12 (16.7)	73 (27.7)	17 (20.5)	11 (23.4)	3 (7.9)	
	Often	85 (14.0)	23 (22.1)	3 (4.2)	29 (11.0)	10 (12.0)	19 (40.4)	1 (2.6)	
	No opinion	19 (3.1)	0	2 (2.9)	9 (3.4)	7 (8.4)	1 (2.1)	0	
Nonionized radiation	Never	476 (78.3)	83 (79.8)	62 (86.1)	201 (76.1)	63 (75.9)	34 (72.3)	33 (86.8)	0.21 (Χ^2^: 19.09)
	Occasionally	82 (13.5)	13 (12.5)	7 (9.7)	40 (15.2)	9 (10.8)	8 (17.0)	5 (13.2)	
	Often	24 (3.9)	5 (4.8)	0	12 (4.5)	3 (3.6)	4 (8.5)	0	
	No opinion	26 (4.3)	3 (2.9)	3 (4.2)	11 (4.2)	8 (9.6)	1 (2.1)	0	
Latex	Never	77 (12.7)	6 (5.8)	12 (16.7)	34 (12.9)	4 (4.8)	5 (10.6)	16 (42.1)	<0.001 (Χ^2^: 61.61)
	Occasionally	105 (17.3)	23 (22.1)	5 (6.9)	44 (16.7)	19 (22.9)	4 (8.5)	10 (26.3)	
	Often	387 (63.7)	66 (63.5)	53 (73.6)	167 (63.3)	53 (63.9)	38 (78.7)	11 (28.9)	
	No opinion	39 (6.4)	9 (8.7)	2 (2.8)	19 (7.2)	7 (8.4)	1 (2.1)	1 (2.6)	
Noise	Never	61 (10.0)	4 (3.8)	9 (12.5)	27 (10.2)	9 (10.8)	6 (12.8)	6 (15.8)	0.367 (Χ^2^: 16.23)
	Occasionally	170 (28.0)	28 (26.9)	18 (25.0)	74 (28.0)	25 (30.1)	17 (36.2)	8 (21.1)	
	Often	339 (55.8)	61 (58.7)	43 (59.7)	148 (56.1)	44 (53.0)	23 (48.9)	20 (52.6)	
	No opinion	38 (6.3)	11 (10.6)	2 (2.8)	15 (5.7)	5 (6.0)	1 (2.1)	4 (10.5)	

Meanwhile, 69.2% (N=421/608) of healthcare workers come into contact with biological materials such as blood, body fluids, and unfixed tissues at work. While more than 70% of doctors, midwives, nurses, and health officers are in contact with biological materials, 53.2% (N=25/47) of health technicians and only 5.3% (N=2/38) of the other group are exposed (p<0.001, Χ2: 90.27). It was observed that exposure to biological materials was high in the majority of workers working in the emergency department (82.2%, N=74/90), laboratory (80.0%, N=32/40), operating room (77.1%, N=81/105), services (77.1%, N=138/179), and intensive care units (71.1%, N=54/76) (p<0.001, Χ2: 85.21).

Additionally, 7.9% (N=48/608) of all healthcare workers stated that they experienced "secondary chemical exposure" during their working lives. Considering the occupational distribution, secondary chemical exposure was highest in midwives (11.1%, N=8/72) and nurses (11.0%, N=29/264).

Evaluation of possible relationships between hazards in the work environment and health conditions of workers

The relationships between the health problems complained of by healthcare workers and their exposure to occupational hazards are evaluated and summarized in Tables 4-5.

**Table 4:** The relationship between workers’ health complaints and their exposure to occupational hazards N: never; Oc: occasionally; Oft: often; N.O.: no opinion; #: Pearson chi-square test The data have been represented as n and %. p<0.05 is considered significant.

Occupational hazards	Exposure frequency	Fertility problems			Renal problems			Liver problems			Dermal problems		
		Yes n (%)	No n (%)	p #	Yes n (%)	No n (%)	p#	Yes n (%)	No n (%)	p#	Yes n (%)	No n (%)	p#
All workers		17 (2.8)	591(97.2)		36 (5.9)	572 (94.1)		41 (6.7)	567 (93.3)		165 (27.1)	443 (72.9)	
Chemotherapeutics	N	14 (2.9)	465 (97.1)	0.569 X^2^:1.13	27 (5.6)	452 (94.4)	0.378 X^2^:1.95	32 (6.7)	447 (93.3)	0.975 X^2^:0.05	119 (24.8)	360 (75.2)	0.077 X^2^:5.13
	Oc	2 (2.1)	94 (97.9)		8 (8.3)	88 (91.7)		7 (7.3)	89 (92.7)		34 (35.4)	62 (64.6)	
	Oft	1 (7.1)	13 (92.9)		0	14 (100.0)		1 (7.1)	13 (92.9)		5 (35.7)	9 (64.3)	
	N.O.	0	19 (100.0)		1 (5.3)	18 (94.7)		1 (5.3)	18 (94.7)		7 (36.8)	12 (63.2)	
Anesthetic gases	N	13 (3.0)	422 (97.0)	0.283 X^2^:2.53	26 (6.0)	409 (94.0)	0.615 X^2^:0.97	22 (5.1)	413 (94.9)	0.003* X^2^:11.37	108 (24.8)	327 (75.2)	0.031* X^2^:6.93
	Oc	4 (4.3)	88 (95.7)		4 (4.3)	88 (95.7)		6 (6.5)	86 (93.5)		35 (38.0)	57 (62.0)	
	Oft	0	61 (100.0)		5 (8.2)	56 (91.8)		10 (16.4)	51 (83.6)		15 (24.6)	46 (75.4)	
	N.O.	0	20 (100.0)		1 (5.0)	19 (95.0)		3 (15.0)	17 (85.0)		7 (35.0)	13 (65.0)	
Aerosolized drugs	N	12 (3.4)	345 (96.6)	0.701 X^2^:0.71	21 (5.9)	336 (94.1)	0.227 X^2^:2.96	16 (4.5)	341 (95.5)	0.009* X^2^:9.32	79 (22.1)	278 (77.9)	0.002* X^2^:12.12
	Oc	4 (2.3)	169 (97.7)		14 (8.1)	159 (91.9)		18 (10.4)	155 (89.6)		57 (32.9)	116 (67.1)	
	Oft	1 (1.8)	54 (98.2)		1 (1.8)	54 (98.2)		7 (12.7)	48 (87.3)		22 (40.0)	33 (60.0)	
	N.O.	0	23 (100.0)		0	23 (100.0)		0	23 (100.0)		7 (30.4)	16 (69.6)	
Sterilization agents	N	4 (1.9)	204 (98.1)	0.391 X^2^:1.88	14 (6.7)	194 (93.3)	0.114 X^2^:4.34	4 (1.9)	204 (98.1)	<0.001* X^2^:19.03	47 (22.6)	161 (77.4)	0.037* X^2^:6.58
	Oc	6 (2.7)	216 (97.3)		8 (3.6)	214 (96.4)		15 (6.8)	207 (93.2)		58 (26.1)	164 (73.9)	
	Oft	7 (4.3)	156 (95.7)		14 (8.6)	149 (91.4)		22 (13.5)	141 (86.5)		56 (34.4)	107 (65.6)	
	N.O.	0	15 (100.0)		0	15 (100.0)		0	15 (100.0)		4 (26.7)	11 (73.3)	
Disinfection agents	N	6 (2.5)	231 (97.5)	0.04* X^2^:6.44	8 (3.4)	229 (96.6)	0.097 X^2^:4.67	10 (4.2)	227 (95.8)	0.079 X^2^:5.08	44 (18.6)	193 (81.4)	<0.001* X^2^:17.76
	Oc	3 (1.4)	205 (98.6)		16 (7.7)	192 (92.3)		17 (8.2)	191 (91.8)		75 (36.1)	133 (63.9)	
	Oft	8 (6.2)	122 (93.8)		10 (7.7)	120 (92.3)		13 (10.0)	117 (90.0)		40 (30.8)	90 (69.2)	
	N.O.	0	33 (100.0)		2 (6.1)	31 (93.9)		1 (3.0)	32 (97.0)		6 (18.2)	27 (81.8)	
Xylene	N	16 (3.5)	442 (96.5)	0.7 X^2^:0.71	27 (5.9)	431 (94.1)	0.569 X^2^:1.13	27 (5.9)	431 (94.1)	0.116 X^2^:4.31	107 (23.4)	351 (76.6)	0.002* X^2^:12.71
	Oc	1 (2.4)	41 (97.6)		3 (7.1)	39 (92.9)		3 (7.1)	39 (92.9)		18 (42.9)	24 (57.1)	
	Oft	0	16 (100.0)		0	16 (100.0)		3 (18.8)	13 (81.3)		8 (50.0)	8 (50.0)	
	N.O.	0	92 (100.0)		6 (6.5)	86 (93.5)		8 (8.7)	84 (91.3)		32 (34.8)	60 (65.2)	
Toluene	N	15 (3.2)	448 (96.8)	0.781 X^2^:0.49	27 (5.8)	436 (94.2)	0.603 X^2^:1.01	27 (5.8)	436 (94.2)	0.041 X^2^:6.37	109 (23.5)	354 (76.5)	<0.001* X^2^:13.82
	Oc	1 (2.5)	39 (97.8)		3 (7.5)	37 (92.5)		3 (7.5)	37 (92.5)		19 (47.5)	21 (52.5)	
	Oft	0	13 (100.0)		0	13 (100.0)		3 (23.1)	10 (76.9)		6 (46.2)	7 (53.8)	
	N.O.	1 (1.1)	91 (98.9)		6 (6.5)	86 (93.5)		8 (8.7)	84 (91.3)		31 (33.7)	61 (66.3)	
Formaldehyde	N	11 (3.0)	360 (97.0)	0.668 X^2^:0.81	21 (5.7)	350 (94.3)	0.503 X^2^:1.38	15 (4.0)	356 (96.0)	<0.001* X^2^:18.40	80 (21.6)	291 (78.4)	<0.001* X^2^:16.09
	Oc	5 (3.9)	122 (96.1)		8 (6.3)	119 (93.7)		14 (11.0)	113 (89.0)		42 (33.1)	85 (66.9)	
	Oft	1 (1.6)	62 (98.4)		6 (9.5)	57 (90.5)		11 (17.5)	52 (82.5)		27 (42.9)	36 (57.1)	
	N.O.	0	47 (100.0)		1 (2.1)	46 (97.9)		1 (2.1)	46 (97.9)		16 (64.0)	31 (66.0)	
Surgical smoke	N	14 (3.3)	404 (96.7)	0.59 X^2^:1.05	22 (5.3)	396 (94.7)	0.584 X^2^:1.08	22 (5.3)	396 (94.7)	0.041 X^2^:6.38	105 (25.1)	313 (74.9)	0.187 X^2^:3.35
	Oc	2 (2.2)	87 (97.8)		6 (6.7)	83 (93.3)		9 (10.1)	80 (89.9)		28 (31.5)	61 (68.5)	
	Oft	1 (1.4)	73 (98.6)		6 (8.1)	68 (91.9)		9 (12.2)	65 (87.8)		25 (33.8)	49 (66.2)	
	N.O.	0	27 (100.0)		2 (7.4)	25 (92.6)		1 (3.7)	26 (96.3)		7 (25.9)	20 (74.1)	
Ionized radiation	N	11 (3.0)	351 (97.0)	0.795 X^2^:0.46	20 (5.5)	342 (94.5)	0.642 X^2^:0.89	20 (5.5)	342 (94.5)	0.119 X^2^:4.25	86 (23.8)	276 (76.2)	0.062 X^2^:5.56
	Oc	3 (2.1)	139 (97.9)		11 (7.7)	131 (92.3)		10 (7.0)	132 (93.0)		47 (33.1)	95 (66.9)	
	Oft	3 (3.5)	82 (96.5)		5 (5.9)	80 (94.1)		10 (11.8)	75 (88.2)		27 (31.8)	58 (68.2)	
	N.O.	0	19 (100.0)		0	19 (100.0)		1 (5.3)	18 (94.7)		5 (26.3)	14 (73.7)	
Nonionized radiation	N	14 (2.9)	462 (97.1)	0.644 X^2^:0.88	28 (5.9)	448 (94.1)	0.599 X^2^:1.03	32 (6.7)	444 (93.3)	0.941 X^2^:0.12	128 (26.9)	348 (73.1)	0.443 X^2^:1.63
	Oc	3 (3.7)	79 (96.3)		7 (8.5)	75 (91.5)		6 (7.3)	76 (92.7)		20 (24.4)	62 (75.6)	
	Oft	0	24 (100.0)		1 (4.2)	23 (95.8)		2 (8.3)	22 (91.7)		9 (37.5)	15 (62.5)	
	N.O.	0	26 (100.0)		0	26 (100.0)		1 (3.8)	25 (96.2)		8 (30.8)	18 (69.2)	
Latex	N	2 (2.6)	75 (97.4)	0.992 X^2^:0.02	3 (3.9)	74 (96.1)	0.536 X^2^:1.25	4 (5.2)	73 (94.8)	0.305 X^2^:2.37	14 (18.2)	63 (81.8)	0.034* X^2^:6.75
	Oc	3 (2.9)	102 (97.1)		5 (4.8)	100 (95.2)		4 (3.8)	101 (96.2)		22 (21.0)	83 (79.0)	
	Oft	11 (2.8)	376 (97.2)		26 (6.7)	361 (93.3)		30 (7.8)	357 (92.2)		116 (30.0)	271 (70.0)	
	N.O.	1 (2.6)	38 (97.4)		2 (5.1)	37 (94.9)		3 (7.7)	36 (92.3)		13 (33.3)	26 (66.7)	
Noise	N	1 (1.6)	60 (98.4)	0.062 X^2^:5.55	2 (3.3)	59 (96.7)	0.639 X^2^:0.89	2 (3.3)	59 (96.7)	0.492 X^2^:1.42	14 (23.0)	47 (77.0)	0.357 X^2^:2.06
	Oc	1 (0.6)	169 (99.4)		11 (6.5)	159 (93.5)		13 (7.6)	157 (92.4)		41 (24.1)	129 (75.9)	
	Oft	14 (4.1)	325 (95.9)		21 (6.2)	318 (93.8)		24 (7.1)	315 (92.9)		99 (29.2)	240 (70.8)	
	N.O.	1 (2.6)	37 (97.4)		2 (5.3)	36 (94.7)		2 (5.3)	36 (94.7)		11 (28.9)	27 (71.1)	

**Table 5:** The relationship between workers’ health complaints and their exposure to occupational hazards N: never; Oc: occasionally; Oft: often; N.O.: no opinion; #: Pearson chi-square test The data have been represented as n and %. p<0.05 is considered significant.

Occupational hazards	Exposure frequency	Allergy			Neurological problems			Lung problems			Varicose vein		
		Yes, n (%)	No, n (%)	p#	Yes, n (%)	No n (%)	p#	Yes, n (%)	No n (%)	p#	Yes, n (%)	No n (%)	p#
All workers		166 (27.3)	442 (72.7)		103 (16.9)	505 (83.1)		88 (14.5)	520 (85.5)		201 (33.1)	407 (66.9)	
Chemotherapeutics	N	121 (25.3)	358 (74.7)	0.141 X^2^:3.92	74 (15.4)	405 (84.6)	0.195 X^2^:3.27	65 (13.6)	414 (86.4)	0.048* X^2^:6.09	149 (31.1)	330 (68.9)	0.016* X^2^:8.29
	Oc	33 (34.4)	63 (65.6)		22 (22.9)	74 (77.1)		17 (17.7)	79 (82.3)		37 (38.5)	59 (61.5)	
	Oft	5 (35.7)	9 (64.3)		2 (14.3)	12 (85.7)		5 (35.7)	9 (64.3)		9 (64.3)	5 (35.7)	
	N.O.	7 (36.8)	12 (63.2)		5 (26.3)	14 (73.7)		1 (5.3)	18 (94.7)		6 (31.6)	13 (68.4)	
Anesthetic gases	N	115 (26.4)	320 (73.6)	0.678 X^2^:0.78	70 (16.1)	365 (83.9)	0.696 X^2^:0.72	58 (13.3)	377 (86.7)	0.303 X^2^:2.39	144 (33.1)	291 (66.9)	0.854 X^2^:0.32
	Oc	27 (29.3)	65 (70.7)		18 (19.6)	74 (80.4)		16 (17.4)	76 (82.6)		30 (32.6)	62 (67.4)	
	Oft	14 (23.0)	47 (77.0)		11 (18.0)	50 (82.0)		12 (19.7)	49 (80.3)		18 (29.5)	43 (70.5)	
	N.O.	10 (50.0)	10 (50.0)		4 (20.0)	16 (80.0)		2 (10.0)	18 (90.0)		9 (45.0)	11 (55.0)	
Aerosolized drugs	N	90 (25.2)	267 (74.8)	0.025* X^2^:7.37	50 (14.0)	307 (86.0)	<0.001* X^2^:16.82	40 (11.2)	317 (88.8)	<0.001* X^2^:15.80	116 (32.5)	241 (67.5)	0.04* X^2^:6.43
	Oc	42 (24.3)	131 (75.7)		30 (17.3)	143 (82.7)		30 (17.3)	143 (82.7)		50 (28.9)	123 (71.1)	
	Oft	23 (41.8)	32 (58.2)		20 (36.4)	35 (63.6)		17 (30.9)	38 (69.1)		26 (47.3)	29 (52.7)	
	N.O.	11 (47.8)	12 (52.2)		3 (13.0)	20 (87.0)		1 (4.3)	22 (95.7)		9 (39.1)	14 (60.9)	
Sterilization agents	N	61 (29.3)	147 (70.7)	0.03* X^2^:7.02	38 (18.3)	170 (81.7)	0.339 X^2^:2.16	26 (12.5)	182 (87.5)	0.108 X^2^:4.45	68 (32.7)	140 (67.3)	0.128 X^2^:4.11
	Oc	46 (20.7)	176 (79.3)		31 (14.0)	191 (86.0)		29 (13.1)	193 (86.9)		64 (28.8)	158 (71.2)	
	Oft	52 (31.9)	111 (68.1)		31 (19.0)	132 (81.0)		32 (19.6)	131 (80.4)		63 (38.7)	100 (61.3)	
	N.O.	7 (46.7)	8 (53.3)		3 (20.0)	12 (80.0)		1 (6.7)	14 (93.3)		6 (40.0)	9 (60.0)	
Disinfection agents	N	53 (22.4)	184 (77.6)	<0.001* X^2^:17.30	29 (12.2)	208 (87.8)	0.002* X^2^:12.74	25 (10.5)	212 (89.5)	0.017* X^2^:8.18	64 (27.0)	173 (73.0)	0.003* X^2^:11.74
	Oc	50 (24.0)	158 (76.0)		35 (16.8)	173 (83.2)		33 (15.9)	175 (84.1)		69 (33.2)	139 (66.8)	
	Oft	54 (41.5)	76 (58.5)		35 (26.9)	95 (73.1)		28 (21.5)	102 (78.5)		58 (44.6)	72 (55.4)	
	N.O.	9 (27.3)	24 (72.7)		4 (12.1)	29 (87.9)		2 (6.1)	31 (93.9)		10 (30.3)	23 (69.7)	
Xylene	N	119 (26.0)	339 (74.0)	0.101 X^2^:4.59	76 (16.6)	382 (83.4)	0.027* X^2^:7.21	65 (14.2)	393 (85.8)	0.031* X^2^:6.97	143 (31.2)	315 (68.8)	0.025* X^2^:7.39
	Oc	12 (28.6)	30 (71.4)		13 (31.0)	29 (69.0)		8 (19.0)	34 (81.0)		19 (45.2)	23 (54.8)	
	Oft	8 (50.0)	8 (50.0)		5 (31.3)	11 (68.8)		6 (37.5)	10 (62.5)		9 (56.3)	7 (43.8)	
	N.O.	27 (29.3)	65 (70.7)		9 (9.8)	83 (90.2)		9 (9.8)	83 (90.2)		30 (32.6)	62 (67.4)	
Toluene	N	122 (26.3)	341 (73.7)	0.563 X^2^:1.15	78 (16.8)	385 (83.2)	0.058 X^2^:5.69	67 (14.5)	396 (85.5)	0.056 X^2^:5.77	145 (31.3)	318 (68.7)	0.01* X^2^:9.11
	Oc	12 (30.0)	28 (70.0)		12 (30.0)	28 (70.0)		7 (17.5)	33 (82.5)		19 (47.5)	21 (52.5)	
	Oft	5 (38.5)	8 (61.5)		4 (30.8)	9 (69.2)		5 (38.5)	8 (61.5)		8 (61.5)	5 (38.5)	
	N.O.	27 (29.3)	65 (70.7)		9 (9.8)	83 (90.2)		9 (9.8)	83 (90.2)		29 (31.5)	63 (68.5)	
Formaldehyde	N	86 (23.2)	285 (76.8)	0.014* X^2^:8.47	56 (15.1)	315 (84.9)	0.09 X^2^:4.81	44 (11.9)	327 (88.1)	0.002* X^2^:12.33	104 (28.0)	267 (72.0)	0.001* X^2^:13.32
	Oc	38 (29.9)	89 (70.1)		30 (23.6)	97 (76.4)		21 (16.5)	106 (83.5)		53 (41.7)	74 (58.3)	
	Oft	25 (39.7)	38 (60.3)		11 (17.5)	52 (82.5)		18 (28.6)	45 (71.4)		29 (46.0)	34 (54.0)	
	N.O.	17 (36.2)	30 (63.8)		6 (12.8)	41 (87.2)		5 (10.6)	42 (89.4)		15 (31.9)	32 (68.1)	
Surgical smoke	N	108 (25.8)	310 (74.2)	0.651 X^2^:0.86	66 (15.8)	352 (84.2)	0.311 X^2^:2.36	56 (13.4)	362 (86.6)	0.154 X^2^:3.75	134 (32.1)	284 (67.9)	0.387 X^2^:1.90
	Oc	27 (30.3)	62 (69.7)		20 (22.5)	69 (77.5)		19 (21.3)	70 (78.7)		35 (39.3)	54 (60.7)	
	Oft	21 (28.4)	53 (71.6)		13 (17.6)	61 (82.4)		12 (16.2)	62 (83.8)		23 (31.1)	51 (68.9)	
	N.O.	10 (37.0)	17 (63.0)		4 (14.8)	23 (85.2)		1 (3.7)	26 (96.3)		9 (33.3)	18 (66.7)	
Ionized radiation	N	90 (24.9)	272 (75.1)	0.153 X^2^:3.75	47 (13.0)	315 (87.0)	0.007* X^2^:9.86	49 (13.5)	313 (86.5)	0.319 X^2^:2.29	114 (31.5)	248 (68.5)	0.551 X^2^:1.19
	Oc	47 (33.1)	95 (66.9)		32 (22.5)	110 (77.5)		21 (14.8)	121 (85.2)		46 (32.4)	96 (67.6)	
	Oft	21 (24.7)	64 (75.3)		20 (23.5)	65 (76.5)		17 (20.0)	68 (80.0)		32 (37.6)	53 (62.4)	
	N.O.	8 (42.1)	11 (57.9)		4 (21.1)	15 (78.9)		1 (5.3)	18 (94.7)		9 (47.4)	10 (52.6)	
Nonionized radiation	N	123 (25.8)	353 (74.2)	0.608 X^2^:1.00	72 (15.1)	404 (84.9)	0.002* X^2^:12.34	66 (13.9)	410 (86.1)	0.007* X^2^:10.05	146 (30.7)	330 (69.3)	0.06 X^2^:5.62
	Oc	24 (29.3)	58 (70.7)		17 (20.0)	65 (79.3)		12 (14.6)	70 (85.4)		34 (41.5)	48 (58.5)	
	Oft	8 (33.3)	16 (66.7)		10 (41.7)	14 (58.3)		9 (37.5)	15 (62.5)		11 (45.8)	13 (54.2)	
	N.O.	11 (42.3)	15 (57.7)		4 (15.4)	22 (84.6)		1 (3.8)	25 (96.2)		10 (38.5)	16 (61.5)	
Latex	N	20 (26.0)	57 (74.0)	0.927 X^2^:0.15	11 (14.3)	66 (85.7)	0.506 X^2^:1.36	10 (13.0)	67 (87.0)	0.706 X^2^:0.70	21 (27.3)	56 (72.7)	0.011* X^2^:9.06
	Oc	30 (28.6)	75 (71.4)		14 (13.3)	91 (86.7)		13 (12.4)	92 (87.6)		23 (21.9)	82 (78.1)	
	Oft	107 (27.6)	280 (72.4)		68 (17.6)	319 (82.4)		59 (15.2)	328 (84.8)		141 (36.4)	246 (63.6)	
	N.O.	9 (23.1)	30 (76.9)		10 (25.6)	29 (74.4)		6 (15.4)	33 (84.6)		16 (41.0)	23 (59.0)	
Noise	N	15 (24.6)	46 (75.4)	0.204 X^2^:3.18	12 (19.7)	49 (80.3)	0.251 X^2^:2.76	12 (19.7)	49 (80.3)	0.31 X^2^:2.34	19 (31.1)	42 (68.9)	0.003* X^2^:11.38
	Oc	39 (22.9)	131 (77.1)		21 (12.4)	149 (87.6)		20 (11.8)	150 (88.2)		39 (22.9)	131 (77.1)	
	Oft	102 (30.1)	237 (69.9)		59 (17.4)	280 (82.6)		49 (14.5)	290 (85.5)		128 (37.8)	211 (62.2)	
	N.O.	10 (26.3)	28 (73.7)		11 (28.9)	27 (71.1)		7 (18.4)	31 (81.6)		15 (39.5)	23 (60.5)	

When those who have never been exposed to chemotherapeutics are taken as a reference, the odds ratios of experiencing respiratory problems and varicosis in those who are frequently exposed are 3.54 (95% CI: 1.15-10.89; p=0.028) and 3.99 (95% CI: 1.31-12.10; p=0.015), respectively. When taken for those who have never been exposed to anesthetic gases as a reference, the odds ratios of liver problems and dermal problems in those often exposed are 3.68 (95% CI: 1.65-8.21; p=0.001) and 1.86 (95% CI: 1.16-2.99; p=0.010), respectively. Using those who were never exposed to sterilization agents in the hospital as a reference, the odds ratio of liver problems in those who were occasionally exposed was 3.70 (95% CI: 1.21-11.32; p=0.022) and in those who were often exposed was 7.96 (95% CI: 2.68-23.59; p<0.001). Similarly, the odds ratio of experiencing dermal problems in those frequently exposed to sterilization agents is 1.79 (95% CI: 1.13-2.84; p=0.013). Considering those who have never been exposed, the odds ratios of allergies, neurological complaints, and respiratory problems in those frequently exposed to disinfectants are 2.47 (95% CI: 1.55-3.92; p<0.001), 2.64 (95% CI: 1.53-4.57; p<0.001), and 2.33 (95% CI: 1.29-4.19; p=0.005), respectively. Like many other hazards, exposure to formaldehyde was found to be significantly associated with various complaints depending on the frequency of exposure: liver problems (for occasionally OR: 2.94 (95% CI: 1.38-6.28); p=0.005; for often OR: 5.02 (95% CI: 2.19-11.52); p<0.001), dermal problems (for occasionally OR: 1.80 (95% CI: 1.15-2.80); p=0.010; for often OR: 2.73 (95% CI: 1.56-4.76); p<0.001). Liver problems are approximately 2.5 times more common in workers often exposed to surgical smoke (OR: 2.49 (95% CI: 1.10-5.65). The odds ratio of neurological problems in those frequently exposed to non-ionizing radiation compared to those who were never exposed was 4.01 (95% CI: 1.71-9.37; p=0.001).

In addition, no statistically significant difference was found between the exposure of workers with children to the examined physical, chemical, and biological occupational hazards (occasionally and often) and the premature birth of their babies (p>0.05).

Evaluation of workers' awareness, attitudes, opinions, and some knowledge levels on OHS

Specifically, 78.5% (N=477/608) of the workers stated that they received training on occupational risks. Among the professions, the highest frequencies of trained workers are midwives, health officers, and nurses (84.5-87.5%), while those with the lowest frequencies of trained workers are doctors with 56.7% (N=59/104). Additionally, 71.7% (N=436/608) of the workers stated that they were aware of the 'worker health and safety committee' in their hospitals. The profession least aware of the existence of this committee was doctors (51.0% (N=53/104) p<0.001, Χ2: 40.05).

When the frequency of healthcare workers knowing about the existence of warnings indicating danger risks in the hospital was examined, 85.4% (N=519/608) of all healthcare workers stated that these warning signs existed, 3.5% (N=21/608) stated that they did not exist, and 11.2% (N=68/608) stated that they had no idea. The professional group with the least awareness of warning signs is doctors (69.2%, N=72/104), while the highest awareness belongs to health technicians (95.7%, N=45/47) (p<0.001, Χ2: 37.86).

As preventive practices regarding biological infections in the hospital, hepatitis B, hepatitis A, and tetanus vaccinations are given during periodic health screenings, and protective practices are provided immediately after a post-contact accident notification. Those who know correctly that these four practices exist constitute only 20.6% (N=125/608) of healthcare workers, and 27.3% (N=72/264) of nurses and 22.2% (N=16/72) of midwives have correct knowledge about these issues, and doctors are the professional group with the least knowledge about infection practices, with 8.7% (N=9/104).

Additionally, 74.7% (N=454/608) of healthcare workers have been vaccinated against hepatitis B, and 17.1% (N=104/608) do not remember whether they have received the vaccine. While 77.1% (N=469/608) of the workers had a tetanus vaccination, 17.8% (N=108/608) did not remember whether they had or not. It was observed that doctors and nurses were vaccinated at the highest frequency, at over 80% (p<0.05). It was found that 94.6% (N=575/608) of all healthcare workers were vaccinated against COVID-19, and there was no difference between the professions in terms of vaccination status (p=0.196, Χ2: 13.52).

In the hospital, waste classification is made for sharp waste, medical waste, hazardous waste, and household waste. It was found that 76.6% (n=466/608) of the employees had correct knowledge about waste classification in the hospital.

Moreover, 8% (N=49/608) of the workers are involved in chemotherapy drug preparation, and 5.9% (N=36/608) are involved in the distribution or application of chemotherapy drugs. During the preparation, only 22.4% (N=11/49) of the workers reported that they always prepared chemotherapeutics in the biosafety cabinet. The frequency of those who always use surgical gloves or examination gloves is 28.6% (N=14/49). Generally, it was determined that approximately 25% of the workers always considered protection. Although the use of many protective equipment such as Luer lock-connected syringes, chemotherapy application sets, face shields, disposable gowns, and chemotherapy gloves has been questioned, the most frequently used protective measures were found as the use of surgical or examination gloves with 55.1% (N=27/49) and the use of protective masks with 42.9% (N=21/49). The protective methods always used by those involved in chemotherapy drug distribution/application are wearing routine uniforms with a frequency of 41.7% (N=15/36), surgical/examination gloves, and chemotherapy application sets with a frequency of 30.6% (N=11/36).

Specifically, 16.9% (N=103/608) of the employees work in a unit that uses ionizing radiation. Men work more frequently in tasks that use ionizing radiation (p<0.001, Χ2: 13.12), and 57.4% (N=27/47) of health technicians and 24% (N=25/104) of doctors work in tasks involving ionizing radiation (p<0.001, Χ2: 74.52). Among the protective equipment that workers always use, lead aprons and thyroid protectors are the most commonly used equipment with a frequency of 28.2% (N=29/103). On the contrary, the least preferred by the workers were lead gloves with 84.5% (N=87/103), dosimeters with 65.0% (N=67/103), and gonad protectors with 61.2% (N=63/103).

When workers' knowledge of hazard symbols used in the workplace is evaluated, the most well-known hazard symbols were flammable (88.3%, N=537/608), explosive (55.6%, N=338/608), biohazard (54.6%, N=332/608), and toxic (50.7%, N=308/608) symbols were also known correctly by approximately half of all workers. However, an irritant (7.9%, N=48/608), gas under pressure (14.8%, N=90/608), and oxidizer (17.3, N=105/608) symbols were found to be the least known danger symbols. While workers' knowledge of hazard symbols does not vary according to profession and gender, the frequency of knowing them correctly is high in those who receive training on OHS. Many of those who are not educated have no idea what the symbols mean (p<0.05).

When the opinions of workers regarding workplace safety were evaluated, it was determined that the majority of all healthcare workers felt safe in the unit they worked in. Doctors were ranked first among the professional groups that did not feel safe in the unit they worked in. The majority of all healthcare workers agree with the statement, "All protective measures are taken against toxic agent exposure in my work area." Among the professions that agree with this opinion, those in the 'other' group and midwives are in the first place with over 70% frequency. However, only 45% of doctors agree with this opinion. The frequency of workers who think that personal protective equipment is adequately provided in the unit they work in is approximately 69%. On the other hand, doctors (20.2%, N=21/104) are the professional group that most frequently thinks that adequate personal protective equipment is not provided. Among all healthcare workers, the frequency of those who fail to use personal protective equipment due to excessive workload was found to be 53.3% (N=324/608).

## Discussion

Determining the prevalence of exposure to various agents at the level of the profession and the units they work in and examining the relationship between possible health problems can contribute to revealing occupational risks for those working in hospitals where many chemicals and physical and biological agents are present.

In this study, 64.1% (N=390/608) of the healthcare professionals are women. It is known that women make up the majority of the workforce in the healthcare sector around the world, and this rate is approximately 70% [6]. Since 70% (N=428/608) of healthcare workers are university graduates and the lowest level of education is high school, unlike many sectors, we studied with a group with a high level of education. The majority of those working in the hospital are nurses (43.4%, N=264/608), followed by doctors (17.1%, N=104/608), health officers (13.7%, N=83/608), and midwives (11.8%, N=72/608). Workers mostly work in the services/wards (29.4%, N=179/608) and secondly in the operating room department (17.3%, N=105/608). Although healthcare professionals are grouped under a single name, hospital workers have a wide variety of professions and duties.

Although healthcare workers are assumed to be healthier than other people because they make healthier lifestyle choices, there is limited information about how healthcare workers' actual health outcomes compare to the general population. It was determined that 32.1% (N=195/608) of all healthcare workers smoked, and contrary to popular belief, no positive behavior was observed in smoking habits in this hospital. In parallel, the number of individuals who use tobacco products every day in the general population in Turkey is 28.3% [7]. The highest smoking rate is in health officers (51.8%, N=43/83), and the least smoking is in doctors (22.1%, N=23/104). It was observed that workers in the emergency and radiology departments had the highest smoking frequency among units. This study reported that 22.9% (N=139/608) of healthcare workers have a diagnosed chronic disease. In univariate analysis, it was found that the profession with the highest number of chronic diseases was among midwives and health officers, but in multivariate analysis, only age was revealed to be a risk factor. Although chronic disease prevalence among healthcare workers in the United States is lower compared to the general population, diseases have been shown to still be common among healthcare workers and to increase over time at a rate similar to the general population [8].

It was found that 9.2% (N=56/608) of all healthcare workers were diagnosed with occupational diseases. In previous studies on the frequency of occupational diseases in healthcare workers in Turkey, the reported frequencies were generally above 50% [9]. On the other hand, in a study conducted in a hospital authorized to diagnose occupational diseases in Turkey, it was found that 2.1% (n=100) of the cases diagnosed with occupational diseases between 2012 and 2018 were healthcare workers [10]. In this study, the frequency of healthcare workers who stated that they had a work accident at least once in their lives was found to be 31.9% (N=194/608). Changes in the accident frequency may vary depending on individual awareness, workers' PPE use attitudes, or work accident reporting behaviors, as well as the creation of safe areas in the hospital. Nurses, health officers, and midwives in the hospital were found to be at higher risk for work accidents/injuries. On the other hand, among working units, emergency rooms, laboratories, and operating rooms were found to be riskier in terms of work accidents. However, it has been observed that work shifts do not affect the frequency of work accidents. In this study, it was determined that work accidents and injuries were more common in women, and this result supports the findings in other studies [11]. In this study, the most common work accidents were soft tissue trauma (needle stick/cut/bruise, etc.) and musculoskeletal system problems. Similarly, musculoskeletal system diseases are the most common accidents and illnesses among healthcare workers all over the world. It often leads to time away from work due to strains and sprains [5]. It has been confirmed by many national and international studies that workers have a high risk of musculoskeletal diseases, especially back pain, and injuries from needle sticks and sharp objects [12,13]. The frequency of exposure to physical violence was 11.0% (N=67/608). There are studies in Turkey that present data consistent with these findings [14,15], and higher frequency cases of physical violence have also been identified [16]. Doctors commonly suffer from physical violence, followed by health officials in the hospital, and the unit where physical violence occurred most frequently was found to be the emergency department. In a study conducted with emergency healthcare workers from 13 countries, it was found that 65% of workers were physically attacked while on duty [17].

The health problem most frequently complained about by healthcare workers was musculoskeletal system problems (71.9%, N=437/608). Additionally, 33.1% (N=201/608) of the workers reported that they experienced varicose veins, 27.3% (N=166/608) reported that they experienced allergic problems, and 27.1% (N=165/608) reported that they experienced skin-related diseases. Long working hours and movements such as lifting, pulling, and carrying during patient care may cause some problems in the musculoskeletal system of the workers, especially when they are performed quickly, repetitively, and challenging, depending on the intensity of the work. Varicose veins, which one in every three workers complains about, were also commonly observed, especially in midwives and nurses. The incidence of varicose veins was higher in women.

Another noteworthy point in the study is that 29.8% (N=76/255) of women with children stated that they had a history of miscarriage at least once in the past. A study conducted among 7,482 nurses in the United States found that exposure to antineoplastic drugs was associated with a twofold increase in the risk of spontaneous miscarriage, especially in early spontaneous miscarriages, and a 3.5-fold increase in the risk in nulliparous women. It has been reported that exposure to sterilization agents increases the risk of late spontaneous abortion by two times [18]. On the other hand, in another study in which 319 existing study results were statistically evaluated to examine the relationship between occupational hazards that physicians may be exposed to and pregnancy, birth, and newborn outcomes, the evidence was generally found to be insufficient, and the need for high-quality studies was stated [19].

When workers were asked what they think are the risks they encounter most, without any guidance, they generally prioritized the risks of infectious diseases and exposure to violence. It is noteworthy that violence is so prominent in terms of risk perception. However, when compared to the question in which the researchers listed the possible hazards in the work environment and asked about the exposure status of the workers, they reported latex use and noise as the most frequently exposed hazards of healthcare workers.

In this study, complaints about respiratory system problems were 3.5 times more common in workers who were frequently exposed to chemotherapeutic agents compared to those who were never exposed. This result is compatible with studies supporting respiratory complaints in oncology nurses [20,21]. Another risk, which is exposure to anesthetics, has been associated with liver problems. Liver problems were found to be 3.7 times more common in workers frequently exposed to anesthetic gases. Liver- and kidney-related biochemical parameters were found to be significantly higher in operating room personnel exposed to inhalation anesthetics in a previous study [22]. NIOSH [23] also reported that exposure to high concentrations of waste anesthetic gases, even for a short period, causes headaches, irritability, fatigue, nausea, drowsiness, and liver and kidney diseases. It has also been observed that there is a significant relationship between dermal problems in workers exposed to anesthetic gases. Cases of dermatitis due to inhaled sevoflurane and isoflurane exposure have been described in the literature, all of which resolved when exposed workers changed their workplace [24]. Disinfection agents have been shown to be another risk factor for respiratory complaints in healthcare workers. It has been determined that frequent exposure to disinfectants increases respiratory problems by 2.3 times supporting previous studies [25,26]. Exposure to surgical smoke is a significant occupational risk in operating rooms, and healthcare technicians and doctors were exposed. In this study, compared to those who were never exposed to surgical smoke, those who were frequently exposed were 2.5 times more likely to experience liver problems. Considering that surgical smoke contains many particles, chemicals (benzene, toluene, xylene, aldehydes, phenol, cresol, etc.), and biological pollutants, the diversity of their health effects emerges. Hepatitis, anemia, headache, asthma, bronchitis, and throat irritation were reported among these general effects [27]. It has been found that neurological problems are four times more likely to occur if healthcare workers are frequently exposed to non-ionizing radiation. Many studies have reported findings such as stress, headache, fatigue, anxiety, decreased learning potential, impairment in cognitive functions, and impaired concentration associated with exposure to non-ionizing radiation. The most obvious biological effect has been focused on the toxic effects that may occur due to the formation of reactive oxygen species, and it has also been shown to cause neural differentiation [28,29]. In this study, the frequency of premature birth was found to be 12.4% (N=48/397) in healthcare workers exposed to occupational hazards. This result is compatible with the premature birth rate (4-16%) reported by WHO for countries in general [6]. However, in our study, no relationship was found between exposure to occupational hazards and history of preterm birth. More comprehensive research is needed on this subject.

It has been determined that approximately 70% of healthcare workers have contact with biological materials. The hepatitis A and B immunizations investigated in this study are within the scope of the vaccination required to be given to workers by health institutions. In addition, the General Directorate of Public Health provides tetanus and COVID-19 vaccines to hospitals. The frequency of hepatitis B, tetanus, and COVID-19 vaccinations among all healthcare workers was 74.7% (N=454/608), 77.1% (N=469/608), and 94.6% (N=575/608), respectively. Doctors and nurses were more likely to be vaccinated.

In this study, 19.4% (N=20/103) of the personnel working with radiation reported that they always used a dosimeter, while 65.0% (N=67/103) reported that they never used it. However, it can also be seen from previous studies that, although the use of dosimeters by healthcare workers is mandatory, they do not use them regularly [30]. Lead aprons and thyroid protectors come to the fore as protective measures for healthcare workers who report being exposed to radiation.

It is very important for workers to know the hazard symbols used in the work environment in order to prevent any toxic effects that may occur. In our study, the most well-known danger symbols are flammable (88.3%, N=537/608). Explosive, biohazard, and toxic symbols are also known correctly by approximately half of all workers. However, the symbols of irritant (7.9%, N=48/608), gas under pressure (14.8%, N=90/608), and oxidizer (17.3, N=105/608) were the least known hazard symbols. There was no difference in knowledge level between professions, but it was observed that having received OHS training created a significant difference in knowledge level.

Limitations of the study

This study is completed with a high participation rate and includes many details of the subject with a detailed survey evaluation. In addition, the inclusion of evaluations both at the level of all healthcare professionals and at their professions and departments level in the same study enabled the subject to be addressed comprehensively. In terms of occupational toxicology, determining occupational hazards and analyzing the relationship between workers' complaints in low- and high-exposure situations shows a different approach from other studies. However, the fact that the data obtained from the study are based on the statements of the workers and do not include a quantitative environmental measurement or biomonitoring creates a limitation in the evaluation of the results.

## Conclusions

Despite workers mainly considering infectious diseases and physical violence as occupational risks, it is found that health workers are primarily exposed to latex and noise in the hospital. Using strategies to reduce noise might increase workers' comfort. In the hospital, the majority of workers suffer from musculoskeletal system problems. However, they also have several health problems, such as varicose veins, allergic issues, and skin-related diseases. Although there are many studies on infectious diseases or musculoskeletal systems in healthcare workers, there is a need for more studies and precautions to be taken regarding other health problems. This study evaluated the relationship between the frequency of exposure to chemical and physical hazards and workers' complaints. The significant relationships found need to be confirmed with further studies on the effects of more specific occupational chemicals and diseases. It is observed that one in every three workers had a work accident in their life. Since health officers and nurses are more prone to accidents, reviewing the risk assessments specific to this professional group and providing additional training will be necessary. Due to the professional majority of nurses in the hospital, their widespread workplace, and their functional closeness to all professional groups, their roles that reach all teams in occupational health prevention can benefit.

It is recommended that dosimeters be encouraged for personnel working with radiation and that additional initiatives be organized to increase doctors' awareness of OHS practices within the hospital. It is essential to provide training in the hospital that emphasizes safety protocols and risks associated with using chemicals and biological agents and to develop a safety culture. Considering that such a large part of the global workforce is employed in this high-risk sector and the predictions that the need for healthcare workers will increase in the future, all risks that will threaten the health of healthcare workers are significant, and any data and initiatives that will contribute to OHS need to be addressed.
